# Analysis of comb-gnawing behavior in *Apis cerana cerana* (Hymenoptera: Apidae)

**DOI:** 10.1093/jisesa/ieae020

**Published:** 2024-02-28

**Authors:** Qingxin Meng, Rong Huang, Hui Li, Xueyang Gong, Dan Yue, Wutao Jiang, Yakai Tian, Kun Dong

**Affiliations:** Yunnan Provincial Engineering and Research Center for Sustainable Utilization of Honeybee Resources, Eastern Bee Research Institute, College of Animal Science and Technology, Yunnan Agricultural University, Kunming 650201, China; Yunnan Provincial Engineering and Research Center for Sustainable Utilization of Honeybee Resources, Eastern Bee Research Institute, College of Animal Science and Technology, Yunnan Agricultural University, Kunming 650201, China; Yunnan Provincial Engineering and Research Center for Sustainable Utilization of Honeybee Resources, Eastern Bee Research Institute, College of Animal Science and Technology, Yunnan Agricultural University, Kunming 650201, China; Yunnan Provincial Engineering and Research Center for Sustainable Utilization of Honeybee Resources, Eastern Bee Research Institute, College of Animal Science and Technology, Yunnan Agricultural University, Kunming 650201, China; Yunnan Provincial Engineering and Research Center for Sustainable Utilization of Honeybee Resources, Eastern Bee Research Institute, College of Animal Science and Technology, Yunnan Agricultural University, Kunming 650201, China; Yunnan Provincial Engineering and Research Center for Sustainable Utilization of Honeybee Resources, Eastern Bee Research Institute, College of Animal Science and Technology, Yunnan Agricultural University, Kunming 650201, China; Yunnan Provincial Engineering and Research Center for Sustainable Utilization of Honeybee Resources, Eastern Bee Research Institute, College of Animal Science and Technology, Yunnan Agricultural University, Kunming 650201, China; Yunnan Provincial Engineering and Research Center for Sustainable Utilization of Honeybee Resources, Eastern Bee Research Institute, College of Animal Science and Technology, Yunnan Agricultural University, Kunming 650201, China

**Keywords:** comb gnawing, comb age, greater wax moth larvae, wax residue

## Abstract

*Apis cerana cerana* exhibits a prominent biological trait known as comb gnawing. In this study, gnawed combs from colonies during different seasons were collected, investigating the comb age and locations of gnawing. Patterns of comb gnawing were recorded, and the effects of 2 factors, namely, comb type and season, on the mass of wax residues and the gnawed surface area were measured. The results revealed that *A. c. cerana* predominantly gnaws combs that have been used for over 6 months, with gnawing concentrated in the brood-rearing area. In the first 3 seasons, significantly higher masses of wax residues and larger gnawed surface areas were found in greater wax moth larvae (GWML)-infested combs compared to newly built and old combs. Also, there were significantly higher masses and areas gnawed by *A. c. cerana* in old combs compared to newly built combs in all 4 seasons. Compared to other seasons, it exhibited significantly higher masses and areas resulting from comb-gnawing in newly built or old combs in winter. However, there were no significant differences in the masses of wax residues and surface areas gnawed in GWML-infested combs across the first 3 seasons. In conclusion, this study documented the impact of comb type and season on the comb-gnawing behavior of *A. c. cerana*, contributing to beekeeping management practices and the current understanding of bee biology.

## Introduction

A bee nest provides a stable environment for various activities of the colony, including information transmission, nestmate recognition, food storage, and brood rearing (Stabentheiner et al. 2002, Oeder and Schwabe 2021). The formation of a nest involves the secretion of flakes of beeswax from workers’ wax glands, modification of them with their mandibles, and modifying the wax to construct vertically oriented combs with regular hexagonal cells (Gallo and Chittka 2018). A brood cell offers space for various physiological activities of the developing bee, including defecation, spinning cocoons, and molting (Jay 1963). Esters and hydrocarbons are the primary components of beeswax that can absorb dark pigments that are produced from the brood’s secretions, turning the comb color brown or black, and the comb color intensity is typically associated with its age (Tulloch 1980, Berry and Delaplane 2001). After multiple generations of worker rearing, a brood cell becomes reduced in size due to the accumulation of cocoons and other materials (Hepburn et al. 2007, Meng et al. 2023). The reduced size of brood cells negatively impacts the morphological characteristics of workers (Alfalah et al. 2012). For instance, the birth weight of workers significantly reduces after 5 generations of rearing in the brood cells (Hu et al. 2023). It is an important indicator of development status, reflecting the size of their external morphological structures (Sam 1991). In apiaries, the use of old combs may result in weaker workers with underdeveloped external morphological features, negatively impacting their foraging abilities and the productivity of the colony (Taha et al. 2021). Furthermore, old combs tend to accumulate harmful substances such as fungi, bacteria, pesticides, and heavy metals (Conti and Botrè 2001, García et al. 2017, Zhang et al. 2023), which can impair various aspects of bee behavior and function, including learning, memory, olfaction, flight navigation, orientation abilities, and immune function (Balbuena et al. 2015, Leonard et al. 2019, Li et al. 2022).

The greater wax moth (GWM), *Galleria mellonella* L. (Lepidoptera: Pyralidae), is a globally distributed honey bee pest (Ellis et al. 2013). Mated adult moths lay eggs in crevices or wax residues at the bottom of beehives. Each female moth can lay hundreds of eggs at a time with a short reproductive cycle, and their population rapidly increases within beehives (Nielsen and Brister 1977). Newly hatched GWM larvae burrow into old combs for feed. They spin silk to create cocoons and construct multiple tunnels while harming developing bees. If the pupae die, their cell cappings are removed by workers, resulting in the formation of “white-headed pupae” (Wang and Ma 2017). In some instances, bees mature but are unable to exit their cells due to the entanglement of their legs in GWM larvae silk (Katariina et al. 2020). GWMs have a severe negative impact on colony development, ranging from hindering colonies’ reproduction to absconding and corresponding significant economic losses to the beekeeping industry (Sohail et al. 2017).

*Apis cerana* exhibits a prominent biological behavior of gnawing on combs. In beekeeping practice, gnaw marks are fairly common on their comb surface, and a lot of wax residues accumulate at the bottom of beehives. Over the past 7 decades, several Chinese scholars have explored the comb-gnawing behavioral habits of *A. c. cerana*. Wang (1958) was the first to provide a biological description of these bees gnawing on combs, highlighting that this behavior primarily occurs in early spring, summer, and early winter. Xie (1975) believed that *A. c. cerana* gnawing on combs is an adaptation developed over a long period in response to the change in their natural environment, which helps the renewal of combs even in the wild. Qian (1982) suggested that *A. c. cerana* comb-gnawing behavior could be a defense strategy against comb-damaging pests like GWMs. Li (1994) proposed that comb*-*gnawing helps overcome the problem of deteriorated and brittle combs, which are suboptimal for brood rearing. However, current research on the comb-gnawing behavior of *A. c. cerana* is mostly qualitative, lacking sufficient data for quantitative analyses that explore factors that may influence the behavior. *Apis cerana*, a crucial pollinator, is vital for maintaining ecological balance, enhancing crop yield and quality, and conserving biodiversity (Rogers et al. 2014, Geslin et al. 2017). Protecting and managing *A. c. cerana* to ensure their reproduction and pollination services is essential for sustainable agriculture and ecosystem health (Decourtye et al. 2019). In this study, we investigated the influence of comb type and seasons on comb-gnawing behavior, mass of wax residues, and surface area gnawed by *A. c. cerana* to better understand their biological habits and provide practical guidance for effective management strategies.

## Materials and Methods

### Investigation of Comb-Gnawing Behavior

A total of 80 combs were collected from the experimental apiary at the Eastern Bee Research Institute of Yunnan Agricultural University, Kunming, China, with 20 combs each from 4 seasons: spring (March), summer (June), autumn (September), and winter (December) in 2022. The way of selecting these combs was: Firstly, the surfaces of these combs showed gnawed marks, indicating that the walls or entire cells had been removed. We then recorded the location of these gnawed marks on the comb (food storage or brood-rearing area). Importantly, we were interested in which location of the comb was gnawed rather than the total number of gnawed marks, even if there were multiple marks on a single comb, as well as this study did not find any comb with gnawed marks that were present in both the food storage and brood-rearing areas. Subsequently, based on the time recorded on the frame when each comb was placed into the hive, we calculated the age of each gnawed comb. Since all the gnawed combs’ ages were ≤ 1 year, we classified their ages into 2 categories using 6 months as a cutoff (including ≤ or > 6 months). Accordingly, each gnawed comb had a specific age and gnawed location. Finally, we compared the proportions of gnawed combs with varying ages (≤ or > 6 months) or locations (food storage or brood-rearing area) across seasons.

### Recording of Comb-Gnawing Pattern

Three types of combs (3 samples of each type) were collected and photographed: newly built combs, old combs with a 1-year age (abbreviated as old combs), and combs infested with greater wax moth larvae (abbreviated as GWML-infested combs, used for 1 year). Next, we established 3 colonies of *A. c. cerana*, each with a strength of 6-full standard Langstroth frames covered with bees. Subsequently, we replaced 3 combs from each colony with 1 comb of each type. Finally, the pattern of comb-gnawing by the bees was observed and imaged.

### Estimation of Wax Residues and Surface Area Resulting From Comb-Gnawing

**Fig. 1.**
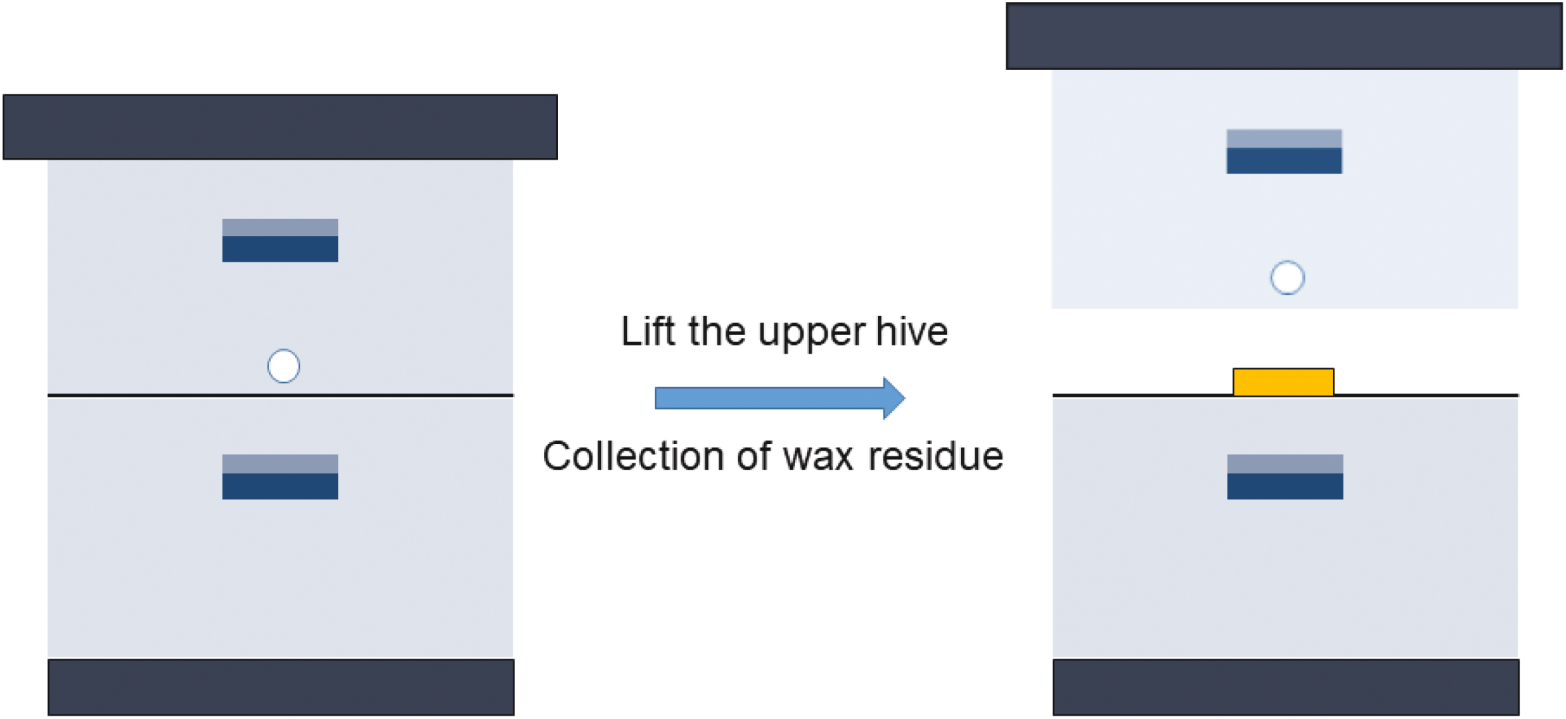
Experimental beehive setup. Beeswax residues were collected in the lower hive body during the study. The upper hive body that served as the brood chamber was lifted to collect wax residue in the collection box.

In the spring (March), 9 colonies were established with 10-frame Langstroth beehives in 3 groups, each containing 3 colonies. Each colony started with a strength of 5-full frames covered with bees and was reared in a brood box with the experimental combs that sat on top of an empty hive body used for collecting wax residues (Fig. 1). In the first group of colonies, all 5 frames contained newly built combs. The second group consisted of 1 old comb and 4 newly built combs. The third group consisted of 1 GWML-infested comb and 4 newly built combs. In every colony, the central comb was the experimental comb. Every 2 days, the brood box was lifted, and the wax residue was collected for mass estimation using an electronic scale. This process was repeated 15 times for each season. In addition, the surface area of the initially introduced experimental combs was measured when the colonies were set up and again after 1 month. This helped to assess the extent of the gnawed comb surface area.

The same methodology was applied to evaluate the influence of newly built and old combs on comb-gnawing behavior during the summer (June), autumn (September), and winter (December) seasons, as well as the effects of GWML-infested combs on comb-gnawing behavior during the summer (June) and autumn (September) seasons.

### Statistical Analysis

Statistical analysis was conducted using GraphPad Prism 9.5 statistical software (GraphPad Software, USA). Initially, the Shapiro–Wilk test was used to examine and confirm that all experimental data followed a normal distribution. Then, differences in the proportions of gnawed combs with varying ages (≤ or > 6 months) or locations (food storage or brood-rearing area) across seasons were compared using t-tests. Subsequently, the two-way ANOVA tests were employed using the main effects-only model to compare the differences in the mass of wax residue or surface area gnawed by *A. c. cerana* regarding the 2 factors: comb type (newly built, old, and GWML-infested combs) and season (spring, summer, autumn, and winter). This ANOVA compared the differences in the data means for each group within 95% confidence intervals, while post hoc Tukey tests were used to correct for multiple comparisons using statistical hypothesis testing. All data are represented as means ± standard error.

## Results

### Characteristics of Gnawed Combs

The results of the investigation on the age and location of gnawed combs during the 4 seasons are shown in Table 1. The *t*-test results indicated that, on average, more combs aged >6 months were gnawed by *A. c. cerana* (17.25 ± 0.63 pieces) than those combs aged ≤6 months (2.75 ± 0.63 pieces) (*t* = 16.30; *df* = 6; *P* < 0.0001). Furthermore, more combs were gnawed in the brood-rearing area (17.75 ± 0.96 pieces) than in the food storage area (2.25 ± 0.96 pieces) (*t* = 22.90; *df* = 6; *P* < 0.0001). This study did not find any comb with gnawed marks that were present in both the food storage and brood-rearing areas.

**Table 1.** Investigation of proportions of gnawed combs with varying ages (≤ or > 6 months) or locations (food storage or brood-rearing area) across 4 seasons

Season	Comb age		Gnawed location	
	≤ 6 months	> 6 months	Food storage	Brood-rearing
Spring (March)	3	17	2	18
Summer (June)	4	16	3	17
Autumn (September)	3	17	3	17
Winter (December)	1	19	1	19

### Pattern of Comb-Gnawing by *A. c. cerana*

**Fig. 2.**
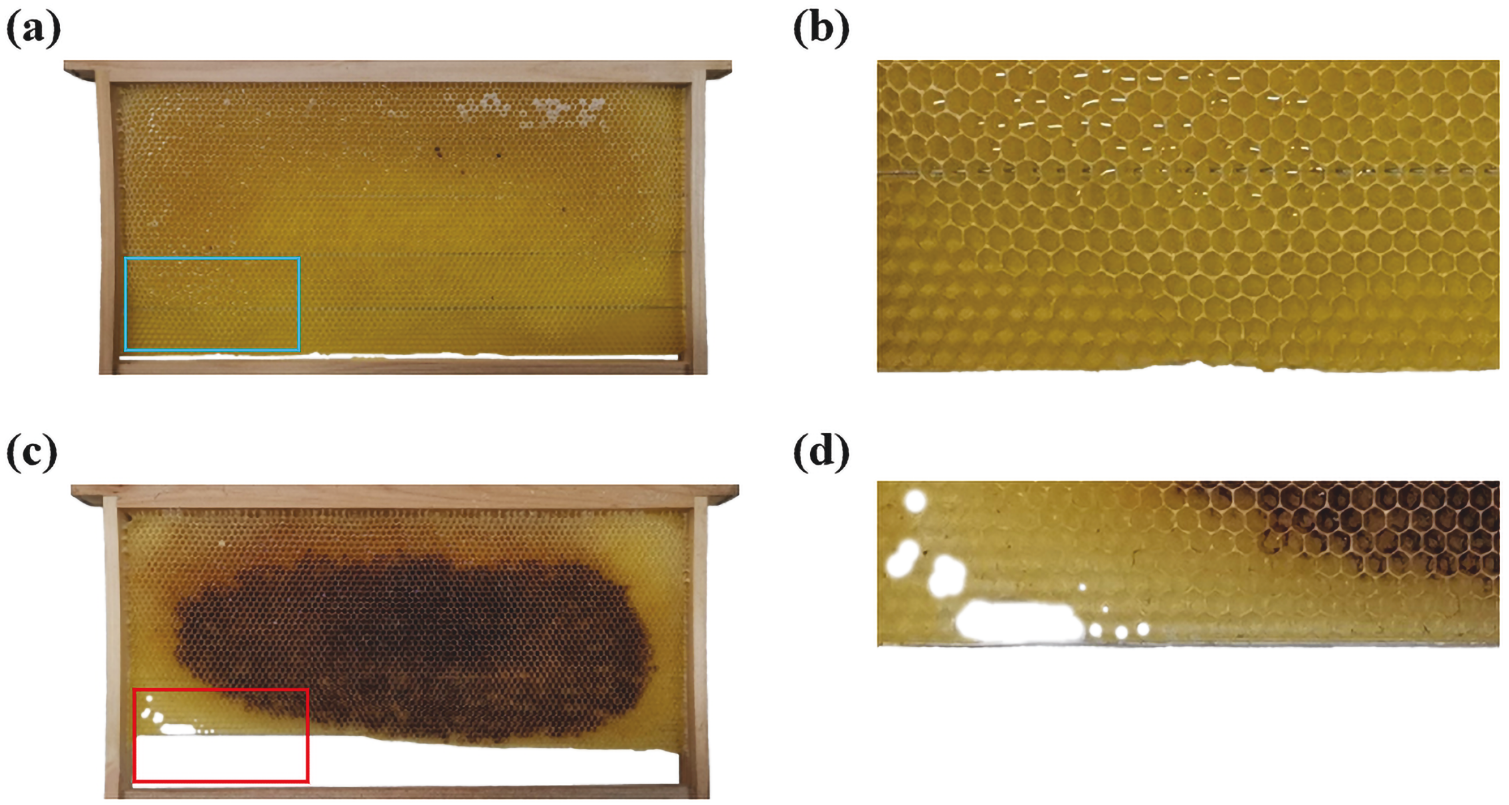
Patterns of *A. c. cerana* gnawing on newly built combs. A) Newly built comb with a foundation that has not been gnawed. B) Enlarged view of the comb marked by the box in A). C) The foundation that had not yet been utilized by the colony was gnawed off. D) Enlarged view of the comb section marked by the box in C).

Due to *A. c. cerana* is usually unable to fully utilize the foundation suitable for standard Langstroth hive frame size, it gnaws on removing the lower edges of the foundation that have not been utilized yet in newly built combs. Specifically, these are the places that have not been raised by the cell walls (Fig. 2A–D).

**Fig. 3.**
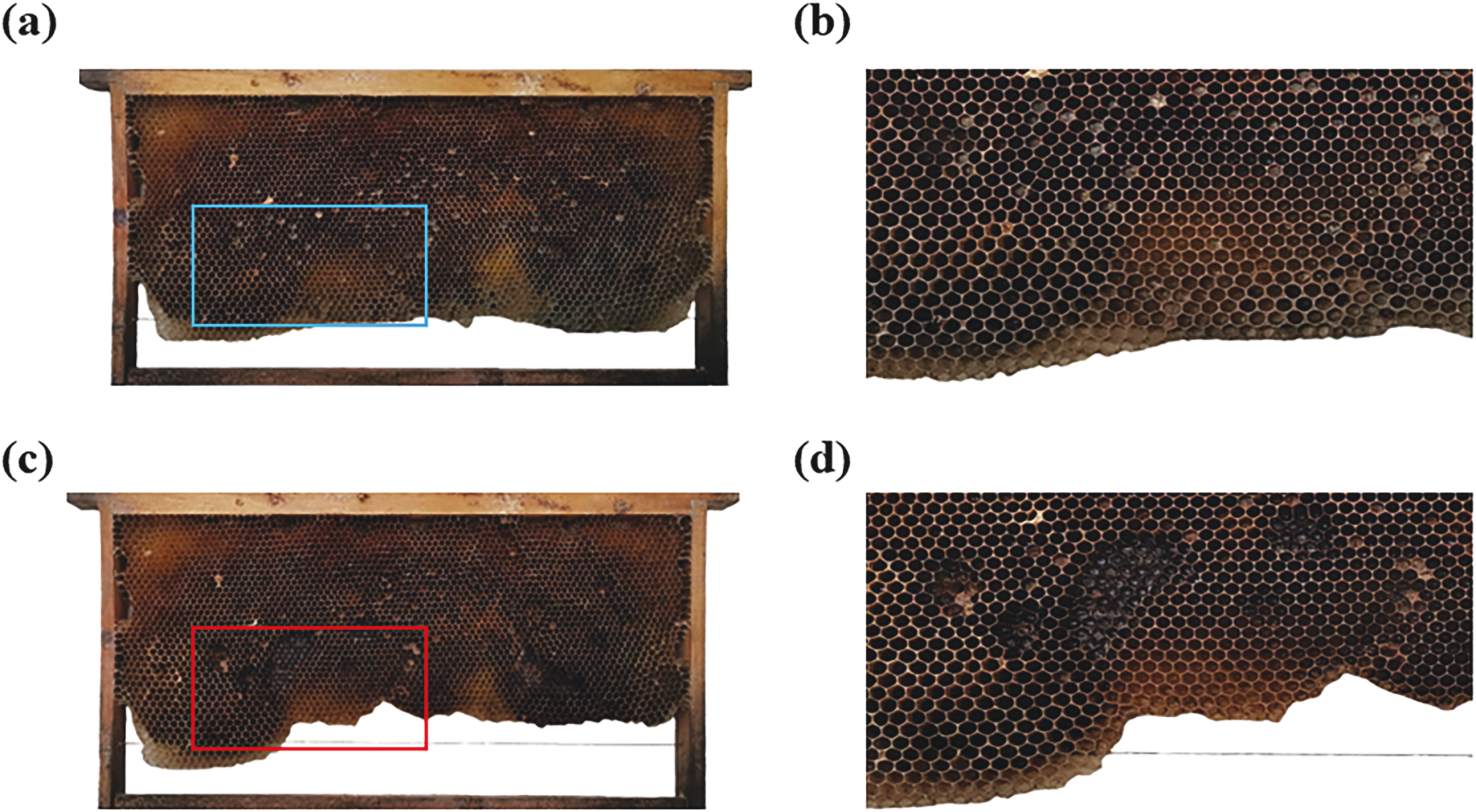
Patterns of *A. c. cerana* gnawing on old combs. A) An old comb with an intact and not yet gnawed brood-rearing area. B) An enlarged view of the comb indicated marked the box in A). C) Old comb with gnawing marks in the brood-rearing area and the lower margin. D) Enlarged view of the comb within the box in C).

In old combs, 2 patterns of comb gnawing by *A. c. cerana* were observed. The first indicated gnawing of the cell walls within the brood-rearing area, leaving the cell base with a small amount of cocoon. The second pattern was the removal of the comb cells in the lower edges of the combs. (Fig. 3A–D).

**Fig. 4.**
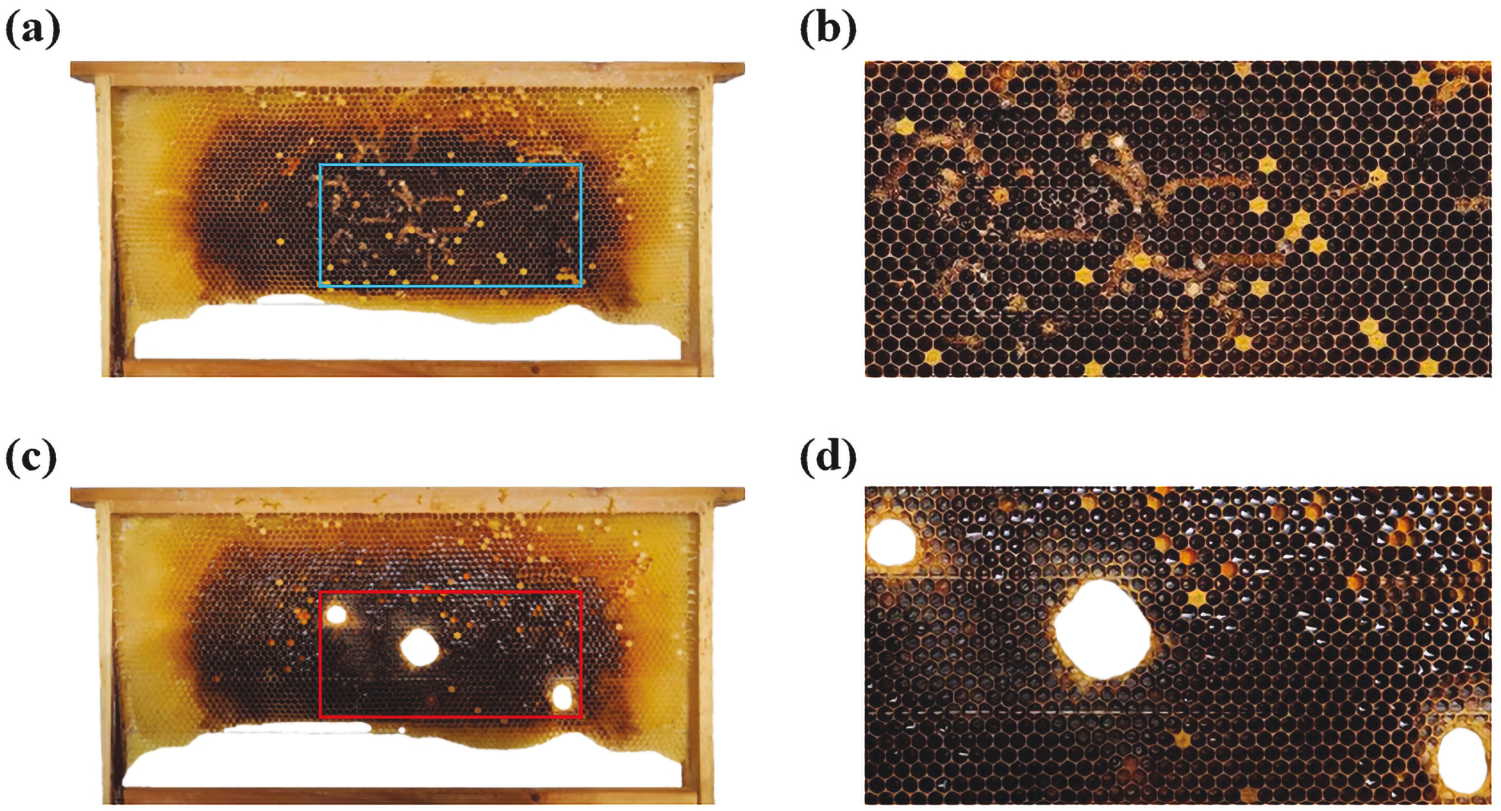
Patterns of *A. c. cerana* gnawing on GWML-infested comb. A) GWML-infested comb that has not yet been gnawed. B) Enlarged view of the comb marked by the box in A). C) Holes in the comb created by bees gnawing away portions of the GWML-infested comb. D) Enlarged view of the comb section indicated by the box in C).

In GWML-infested combs, the bee gnaws away portions of the comb that had GWML spinning silk to create cocoons, resulting in comb holes of various sizes (Fig. 4A–D).

### Comparison of the Mass of Wax Residues and Surface Area Resulting From Comb-Gnawing in Different Types of Combs Across 4 Seasons

**Fig. 5.**
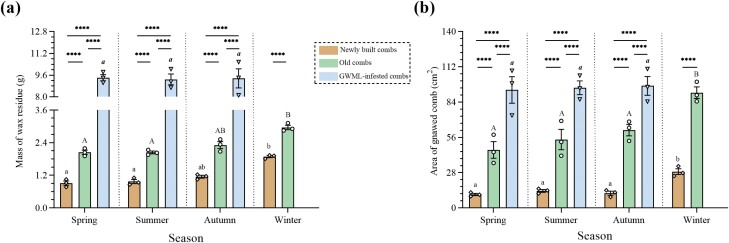
Comparison of the A) mass of wax residues and B) surface area gnawed by *A. c. cerana* in newly built, old, and GWML-infested combs across the 4 seasons. As shown in the graph, * indicates comparisons of gnawing characteristics between different comb types within the same season; lowercase nonbold letters denote comparisons among newly built combs in different seasons; uppercase letters represent comparisons among old combs in different seasons; lowercase bold letters indicate comparisons among GWML-infested combs in different seasons. Data are represented as means ± SE, ****P < 0.0001.

*Comparison of the mass of wax residues gnawed by A. c. cerana*. The comb type significantly affected the mass of wax residues gnawed by *A. c. cerana* (*F*_(2, 27)_ = 1,069, *P* < 0.0001). In the first 3 seasons, the mass of gnawed wax residues was significantly higher for GWML-infested combs (spring: 9.41 ± 0.23 g; summer: 9.28 ± 0.40 g; autumn: 9.36 ± 0.71 g) than for newly built and old combs (spring: 0.91 ± 0.08 g and 2.05 ± 0.08 g, *P* < 0.0001; summer: 0.97 ± 0.07 g and 2.04 ± 0.05 g, *P* < 0.0001; autumn: 1.15 ± 0.05 g and 2.31 ± 0.13 g, *P* < 0.0001). Furthermore, in all 4 seasons, the mass of gnawed wax residues was significantly higher in old combs (winter: 2.96 ± 0.08 g) than in newly built combs (winter: 1.89 ± 0.04 g, *P* < 0.0001). Similarly, the season also significantly affected the mass of gnawed wax residues (*F*_(3, 27)_ = 6.599, *P* = 0.0017). In winter, it exhibited a significantly higher mass of gnawed wax residue in newly built or old combs compared to spring (*P* = 0.0210 and *P* = 0.0210, respectively) and summer (*P* = 0.0159 and *P* = 0.0159, respectively). In contrast, there was no significant difference in GWML-infested combs across the first 3 seasons (*P* > 0.05) (Fig. 5A).

*Comparison of the surface area gnawed by A. c. cerana.* The comb type significantly influenced the gnawed surface area (*F*_(2, 27)_ = 176, *P* < 0.0001). In the first 3 seasons, the gnawed surface area was significantly higher for GWML-infested combs (spring: 93.70 ± 10.56 cm^2^; summer: 95.37 ± 5.46 cm^2^; autumn: 96.87 ± 7.49 cm^2^) than for newly built and old combs (spring: 10.73 ± 0.90 cm^2^ and 46.10 ± 6.55 cm^2^, *P* < 0.0001; summer: 13.57 ± 1.11 cm^2^ and 54.33 ± 8.03 cm^2^, *P* < 0.0001; autumn: 11.73 ± 1.59 cm^2^ and 65.80 ± 6.62 cm^2^, *P* < 0.0001). Furthermore, in all 4 seasons, the gnawed surface area was significantly higher in old combs (winter: 91.43 ± 4.61 cm^2^) than in newly built combs (winter: 28.80 ± 2.31 cm^2^, *P* < 0.0001). Similarly, the season also significantly affected the gnawed surface area (*F*_(3, 27)_ = 11.21, *P* < 0.0001). In winter, it exhibited a significantly higher mass of gnawed surface area in newly built or old combs compared to spring (*P* = 0.0004, and *P* = 0.0004 respectively), summer (*P* = 0.0028 and *P* = 0.0028, respectively), and autumn (*P* = 0.0081 and *P* = 0.0081, respectively). In contrast, there was no significant difference in GWML-infested combs across the first 3 seasons (*P* > 0.05) (Fig. 5B).

## Discussion

### Gnawing Behavior of *A. c. cerana* for Old Combs

The continuous accumulation of excrement, cocoons, and other materials produced during the development of successive generations of broods cannot be completely removed by adult workers (Zhang et al. 2010). This leads to the gradual buildup of these substances within the brood cells, causing structural changes and a reduction in volume inside the cell in old combs (Hepburn et al. 2007). Old combs negatively impact the external morphological dimensions of workers (Hu et al. 2023), the lifespan of honey bees (Berry and Delaplane 2001), colony population and productivity (Dizaji et al. 2008, Taha and Al-Kahtani 2020), and, in turn, the quality of bee products (Rocha et al. 2010). A study examining the brood cell structure of Western honey bees (*A. mellifera*) reported that workers expand the volume of brood cells by carefully cleaning the accumulated debris within the cells and secreting wax to increase the cell wall height, which helps the normal development of broods (Таранов 1961). In contrast, *A. c. cerana* has evolved biological traits to gnaw away old combs, balancing the negative impact of old brood cells on the colony (Meng et al. 2023). In this study, we observed that age is one of the major factors contributing to the comb-gnawing behavior. The mass of wax residue and surface area resulting from comb-gnawing on old combs are significantly higher than those on newly built combs, resulting in the mass of wax residue at the hive bottom significantly increasing when nests have old combs. Additionally, we observed that *A. c. cerana* primarily gnaw cells in the central brood-rearing areas of old combs. Compared to the edges, the higher and more stable temperature of the comb’s central region is more suitable for the queen to lay eggs (Becher and Moritz 2009). This area probably reared more worker generation, with a greater reduction in cell volume, leading the brood-rearing area to show earlier signs of gnawing. Notably, old combs also act as natural repositories for pollutants; with an increasing number of brood generations, the accumulation of substances (such as cocoon and beeswax) capable of enriching pollutants within the combs increases, leading to the contaminations like heavy metals and pesticides enrichment in old combs (Feldhaar and Otti 2020, Monchanin et al. 2021, Scivicco et al. 2022). However, further experimentation is necessary to confirm the relationship between comb-gnawing behavior and pollutant enrichment in old combs.

### Gnawing of GWML-Infested Combs by *A. c. cerana*

The GWM is a widely distributed honey bee pest and also a significant factor influencing comb-gnawing by *A. c. cerana*. We found that they produce significantly more wax residue in terms of mass and surface area when gnawing on GWML-infested combs compared to newly built or old combs. Li et al. (2019) found that *A. c. cerana* selectively ignores adult GWMs, allowing their entry from beehives to lay eggs in crevices and wax residues (Ellis et al. 2013). If a colony predominantly contains old combs, comb-gnawing results in a greater accumulation of wax residue at the bottom of the hive. This concealing environment is highly suitable for oviposition by adult GWMs and the hatching of GWM larvae (Yang et al. 2016b). After hatching, GWM larvae crawl onto a comb where they search for proteins, nectar, pollen, accumulated excrement, and other substances within brood cells that serve as sources of nutrients for their growth and development (Kwadha et al. 2017). Moreover, GWM larvae in combs stimulate comb-gnawing by workers, which results in debris that provides food for late-instar larvae and creates a concealed environment for pupation (Yang et al. 2016a). Several studies suggest that *A. c. cerana* ignores GWM eggs, larvae, pupae, and adults, but they are highly sensitive to the silks and cocoons spun by GWM larvae on the comb and gnaw off the affected comb area. This reduces the threat posed by GWM larvae to the colony. However, even after *A. c. cerana* gnaw on the GWML-infested combs, the larvae that fall into the bottom of the hive continue their life cycle. Therefore, merely gnawing on the infested areas of the comb does not fully eliminate the threat of GWMs to *A. c. cerana*. Consequently, in the beekeeping management of *A. c. cerana* colonies, it is essential to regularly clean the wax residues on the bottom of the beehive to reduce suitable rearing areas for GWMs.

### Influence of Seasons on Comb-Gnawing Behavior of *A. c. cerana*

We found that the comb-gnawing behavior of *A. c. cerana* exhibits seasonal variations. This experiment was conducted in Kunming, Yunnan, China. This region experiences a subtropical-highland monsoon climate with long sunshine hours, a short frost period, and an annual average temperature of 15 °C. During the experiment, the average temperatures for the respective seasons were as follows: Spring (March) 18 °C, Summer (June) 21 °C, Autumn (September) 20 °C, and Winter (December) 10 °C. The behavioral activities of the honey bees are influenced by external temperatures (Abou-Shaara et al. 2017). Across the 4 seasons, the gnawing behavior of *A. c. cerana* exhibited the mass of wax residues and gnawed surface area increasing with the temperature reducing for both newly built and old combs. In winter, when the temperature is lowest, *A. c. cerana* reduces foraging activities and forms clusters to regulate the temperature. Wang (1958) found that the bottom of the hive had more wax residues at this time and that some of the lower edges of the combs had been gnawed off. Interestingly, in winter, *A. c. cerana* creates significantly higher masses of wax residues and gnawed surface areas on old combs than in newly built combs, indicating that even at low temperatures, it continues to make selective choices when gnawing comb. This behavior may serve as an early preparation for wax secretion and the natural replacement of the old comb with a new comb in the spring. Moreover, during winter, when temperatures are lower, GWM larvae enter diapause (Godlewski et al. 2001). We observed no naturally GWML-infested combs during winter and, therefore, could not study comb-gnawing at that time of the year. Based on the seasonal and selective differences in comb-gnawing behavior by *A. c. cerana*, we recommend that beekeepers should replace the old combs with newly built combs and clean the beehive before the onset of the winter season. This practice plays a crucial role in reducing the wax residue produced by comb-gnawing during winter, promoting colony reproduction in the following spring, and effectively preventing GWM infestations.
